# Role of GABA_A_ receptor depolarization-mediated VGCC activation in sevoflurane-induced cognitive impairment in neonatal mice

**DOI:** 10.3389/fncel.2022.964227

**Published:** 2022-09-13

**Authors:** Shuang Zeng, Ruilou Zhu, Yangyang Wang, Yitian Yang, Ningning Li, Ningning Fu, Mingyang Sun, Jiaqiang Zhang

**Affiliations:** ^1^Department of Anesthesiology and Perioperative Medicine, People's Hospital of Zhengzhou University, Henan Provincial People's Hospital, Zhengzhou, China; ^2^Academy of Medical Sciences of Zhengzhou University, Zhengzhou, China

**Keywords:** GABA_A_R, VGCCs, calcium concentration, cognitive impairment, inflammation

## Abstract

**Background:**

In neonatal mice, anesthesia with sevoflurane depolarizes the GABA Type A receptor (GABA_A_R), which leads to cognitive impairment. Calcium accumulation in neurons can lead to neurotoxicity. Voltage-gated calcium channels (VGCCs) can increase intracellular calcium concentration under isoflurane and hypoxic conditions. The underlying mechanisms remain largely unknown.

**Methods:**

Six-day-old mice were anesthetized with 3% sevoflurane for 2 h/day for 3 days. The Y-Maze, new object recognition (NOR) test, the Barnes maze test, immunoassay, immunoblotting, the TUNEL test, and Golgi–Cox staining were used to assess cognition, calcium concentration, inflammatory response, GABA_A_R activation, VGCC expression, apoptosis, and proliferation of hippocampal nerve cells in mice and HT22 cells.

**Results:**

Compared with the control group, mice in the sevoflurane group had impaired cognitive function. In the sevoflurane group, the expression of Gabrb3 and Cav1.2 in the hippocampal neurons increased (*p* < 0.01), the concentration of calcium ions increased (*p* < 0.01), inflammatory reaction and apoptosis of neurons increased (*p* < 0.01), the proliferation of neurons in the DG area decreased (*p* < 0.01), and dendritic spine density decreased (*p* < 0.05). However, the inhibition of Gabrb3 and Cav1.2 alleviated cognitive impairment and reduced neurotoxicity.

**Conclusions:**

Sevoflurane activates VGCCs by inducing GABA_A_R depolarization, resulting in cognitive impairment. Activated VGCCs cause an increase in intracellular calcium concentration and an inflammatory response, resulting in neurotoxicity and cognitive impairment.

## Introduction

With the continued development of surgical and anesthetic techniques, complex surgeries in neonates and infants are increasing year by year, with children needing long-time exposure to systemic narcotic drugs, a key stage in the neuromaturation period, and existing relevant research suggests that babies who receive general anesthesia have a significantly increased risk of abnormal cognition and behavioral science in adulthood (Wilder et al., 2009; Yu et al., 2013; Lin et al., 2017). The US Food and Drug Administration (FDA) issued a drug safety advisory stating that repeated or prolonged use of general anesthesia or sedatives during surgical or medical procedures increases the risk of impaired brain development in infants and children under the age of three and in pregnant women in their third trimester. According to the New England Journal of Medicine, February 2017, repeated and prolonged exposure to general anesthesia in infancy and early childhood remains a neurodevelopmental risk factor (Andropoulos and Greene, 2017). Although current clinical studies demonstrated that general anesthesia has no significant effect on intellectual development in children, other scientists are calling for further research to assess the long-term effects of anesthetics on neurodevelopment, which is vital for clinical decision-making about surgery and anesthesia in children (McCann and Soriano, 2019; Ing et al., 2022).

Among these age-dependent mechanisms, depolarizing/excitatory GABA Type A receptor (GABA_A_R) signal is the primary substrate for other inhibitory effects of GABA anesthetics at more mature ages (Farrant and Kaila, 2007; Nunez and McCarthy, 2008; Feller et al., 2010; Dehorter et al., 2012; Ben-Ari, 2013; Kotani and Akaike, 2013; Antkowiak and Rudolph, 2016). GABA_A_R signaling is mainly excitatory in rostral brain regions in early life due to elevated levels of intraneuronal Cl- maintained by relatively low and high levels of K-2Cl- (KCC2) Cl- exporter and Na-K-2Cl- (NKCC1) Cl- importer, respectively (Dzhala et al., 2005; Farrant and Kaila, 2007; Nunez and McCarthy, 2008). GABA_A_R signaling is gradually inhibited in rodents during the second postnatal week and in humans during the first year of life, primarily due to age-dependent increases in KCC2 (Kontou et al., 2021; Gao et al., 2022). The magnitude of the excitatory GABA_A_R signal and the appropriate time for its transition from excitability to inhibition are critical for normal brain development and function.

Voltage-gated calcium channels (VGCCs) play an important role in the pathway of extracellular Ca^2+^ influx. The regulation of Ca^2+^ influx using VGCCs will trigger a series of cellular reactions. After the release of GABA into mature neurons, GABA_A_R causes Cl^−^ influx and cell membrane hyperpolarization and inhibits VGCCs (Oh et al., 2016). Long-term exposure to sevoflurane results in GABA_A_R activation in developing neurons. VGCCs play an important role in the proliferation and dendrite formation of nerve cells, and an external intervention may affect the development of normal neural circuits (Toth et al., 2016; Agosti et al., 2017). In aging neurons, Ca^2+^ influx increases during depolarization, which increases cytoplasmic Ca^2+^ concentration and leads to excitotoxicity. A previous study suggested that prolonged exposure to the inhaled anesthetic isoflurane can cause a large influx of Ca^2+^ into nerve cells, resulting in cytotoxicity and a decrease in the proliferation rate of neural progenitor cells (Zhao et al., 2013). Nevertheless, there are few studies on the effects of sevoflurane on VGCCs in neurons. The purpose of this study was to understand the effects of sevoflurane on VGCCs in developing hippocampal neurons and to investigate whether it causes long-term cognitive dysfunction in neonatal mice *via* VGCCs.

## Materials and methods

### Mice

All experimental procedures involving mice were approved by the Grass-roots Ethics Review Committee of Zhengzhou University. Efforts were made to minimize the number of mice used in these studies. Pregnant mice (C57BL) were purchased from Charles River Laboratories. The mice were housed at the Animal Center of Zhengzhou University. All mice were fed standard rodent food and water and housed (one mouse per cage) in a controlled environment at 37°C with 12-h light/dark cycles (lights on from 07:00 to 19:00). The day of birth was designated as P0. Young mice were randomly assigned to different groups. Each group included a similar number of female and male mice. We randomly assigned mice to four groups: (1) control plus vehicle; (2) sevoflurane plus vehicle; (3) sevoflurane plus bicuculline; and (4) sevoflurane plus nifedipine. Mice did not experience unexpected lethality and were euthanized according to institutional animal care and committee guidelines.

### Anesthesia and treatment

Multiple surgeries and anesthesia exposure in children are associated with an increased risk of perioperative cognitive impairment. We treated young mice with sevoflurane three times to not only conceptually simulate multiple exposures to anesthesia and surgery in children but also to determine the role of non-surgical anesthesia in the neurocognitive impairment observed in young mice. Specifically, we anesthetized young mice with 3% sevoflurane plus 30% oxygen for 2 h at P6, P7, and P8, which caused no obvious changes in the values of pH, PO_2_, and PCO_2_ in the mice. Control mice received 30% oxygen at the same flow rate in similar chambers and were isolated from their mother. While administering anesthesia with sevoflurane, we continuously monitored sevoflurane and oxygen concentrations. Bicuculline (0.25 mg/kg) (Maejima et al., 2018) and nifedipine (10 mg/kg) (Bernardi et al., 2014) were injected subcutaneously 30 min before anesthesia with sevoflurane. We chose these effective doses of bicuculline, bumetanide, and nifedipine reported in the literature. Immediately after control conditions or anesthesia with sevoflurane, mice were decapitated to obtain the hippocampus at P8 to measure Gabrb3 and Cav1.2 and at P56 to measure dendritic spine density. A separate cohort of mice was used in the Y-Maze, the new object recognition (NOR) test, and the Barnes maze test.

### Y-maze

The rodents were adapted to the room for 30 min prior to testing (the researcher can stay in the room with the lights on but should remain silent during this time). The Y-maze was cleaned with an animal-safe sanitizing solution, and all sanitizing solutions were wiped off with paper towels. It was ensured that the maze was dry. The three arms of the Y-maze were divided into new arms, starting arms, and other arms. The experiment consisted of two stages. The first stage was a training period, in which the new arm was blocked with a partition, and the mice were placed in the initial arm and allowed to move freely in the initial arm and other arms for 10 min. After training, the mice were put back into the cage. Phase two was initiated after an hour. In the second stage, the new arm baffle was removed, and the mice entered through the initial arm and moved freely in all three arms for 5 min. The time and shuttle times of each arm were recorded in 5 min.

### New object recognition test

The NOR test was performed at P58 and P59. In the first trial, a mouse was placed in a square arena (40 cm × 40 cm × 40 cm, with an even lighting of 20 lux) to habituate it for 5 min. We used a black container to cover the mouse. Two identical objects (same shape and color) were placed in opposite corners of the upper half of the area, and then, the mouse was released from the container. We recorded mouse activity and the time spent interacting with the object for 5 min. We repeated the first trial 1 h later, but one of the objects was replaced by a novel object (different shapes and colors). The interaction time with the novel object (novel time) and familial object (familiar time) was recorded separately. The discrimination index was defined as the ratio of novel time to novel time plus familial time, indicating the ability to recognize new objects.

### Barnes maze test

In the Barnes maze experiment, mice were kept in light or dark conditions, and the characteristics of mice were used to test spatial memory ability. Barnes maze by tasteless height is 140 cm, peripheral containing 20 equidistance circular hole, 122 cm diameter circular platform, including the target hole connected to the camera obscura, identify the target hole in mice after it can enter camera obscura hiding, other holes are empty, mice cannot hide after entering. Target identification markers set the space over the hole. The Barnes maze experiment was started on 58 days after birth. The Barnes maze experiment includes three stages: the training stage and the short-term and long-term memory testing stages. The training stage involves training two times a day for 4 consecutive days, with an interval of 2 h every time. Before the Barnes maze experiments, the mice were placed in the test room for 30 min and were allowed to adapt to the environment in the testing room. Before the beginning of each mouse experiment, mice feces were cleaned, and all the experimental apparatuses were cleaned using 75% alcohol and left to evaporate completely before the experiment. The use of a 20-cm high diameter and a 27-cm black plastic barrel limited the mice to the center of the labyrinth Barnes platform, and it took the mice 5 s to make it to the black plastic barrel. The mice remained on the Barnes maze platform until the target box was found, after which the mice would remain in the black case for 1 min after the return to their original cage. The mice would freely search for the “black” box on the Barnes maze platform, and if the mice did not find the target box after 150 s, the experimenter would guide the rats into the target box and keep them in the black box for 30 s. For 4 days after the training phase, the mice would be in the short-term testing stage 24 h after the training for the short-term spatial memory test (short-term retention) and 8 days after the end of the training for the long-term spatial memory test (long-term retention). Smart software was used to identify the target track mice box and enter the black time.

### Brain tissue harvest, lysis, and protein quantification

**Figure 1:**
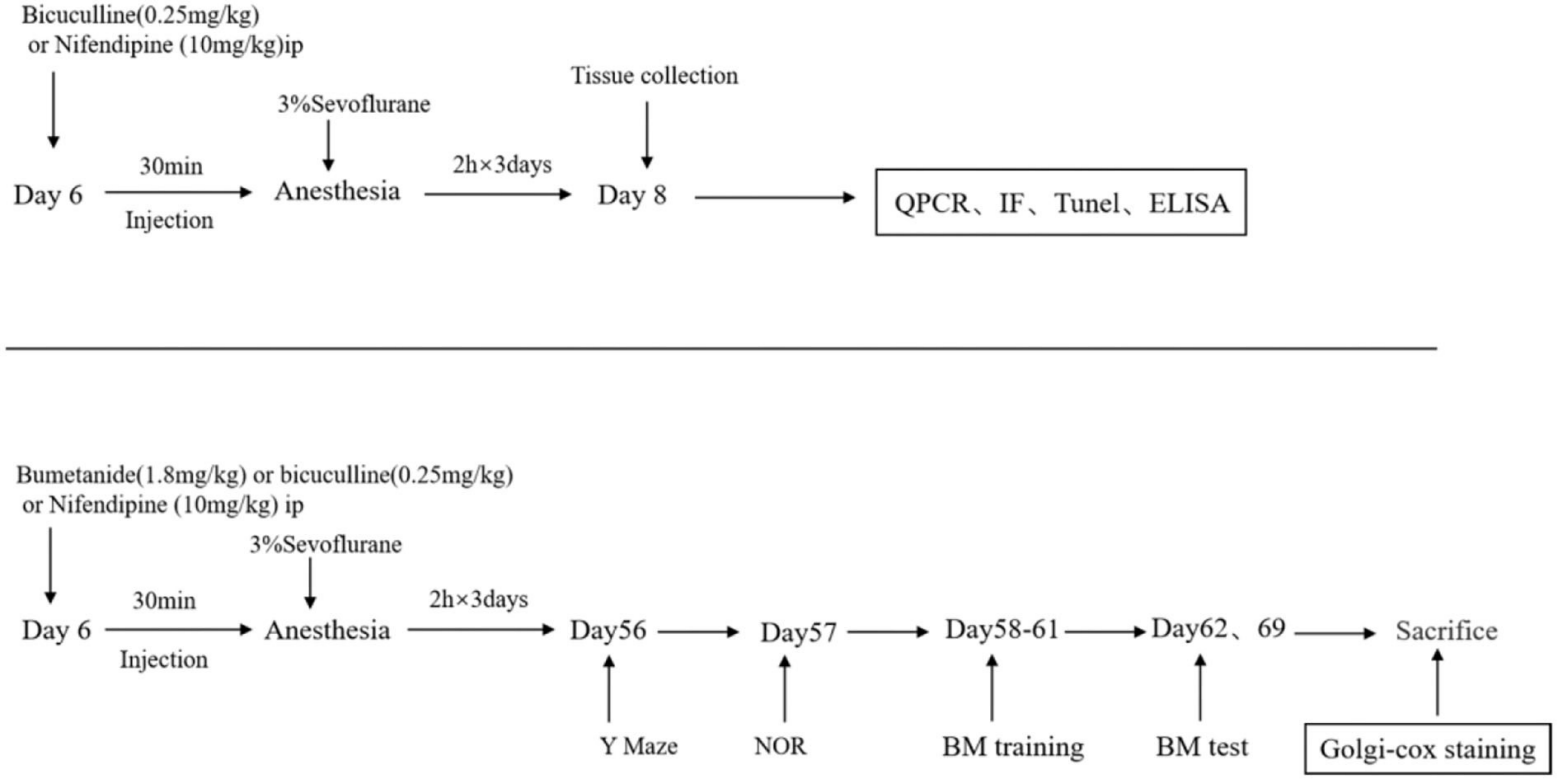
Schematic timeline of an experimental procedure. The mice were treated with bicuculline (a GABA inhibitor, 0.25 mg/kg) or nifedipine (a voltage-gated calcium channel (VGCC) antagonist, 10 mg/kg) intraperitoneally (hype) before being exposed to anesthesia and sevoflurane for 2 h for 3 consecutive days. After the third anesthesia, brain tissues were harvested for western blot and immunofluorescence detection. Behavioral tests were performed on day 56, and mice performed in an open field the new object recognition test (NOR), the Barnes maze training test, and the Barnes maze test. After the Barnes maze tests, the brains of mice were collected for western blot and Golgi–Cox staining.

Mice were decapitated at P8 or P70, and the hippocampus was harvested for QPCR and other experiments (Figure 1).

### Cell transfection

The design and synthesis of small interfering RNA (siRNA) for Gabrb3 and Cav1.2 were completed by Genechem (Shanghai, China). According to the manufacturer's instructions, the transfection assay was conducted using lipofectamine 3000 (Invitrogen, Carlsbad, USA) at a transfected concentration of 50 nM. It should be noted that the transfection should be performed before the inhalation of sevoflurane.

### Exposure to sevoflurane

At 48 h after cell transfection, HT22 cells in six-well plates (1 × 10^6^ cells/well) were exposed to 3% sevoflurane for 6 h at 37°C.

### RT-qPCR

Hippocampal tissues and HT22 cells were treated with the TRIzol reagent for total RNA isolation. For the synthesis of complementary DNA, we performed a reverse transcription reaction on the extracted RNA following the reverse transcription kit instructions.

### Enzyme-linked immunosorbent assay

To detect the contents of interleukin-1β (IL-1β) and interleukin 6 (IL-6) in hippocampal tissues and HT22 cells, we purchased the enzyme-linked immunosorbent assay (ELISA) test kit from Xinbosheng. Before that, hippocampal tissues and cultured cells were used to obtain supernatants by centrifugation at 16,000 × g. Finally, the optical density was quantified at the absorbance of 450 nm using the ELx800 automatic microplate reader (BioTek, Winooski, USA).

### Immunofluorescence staining

Neuronal cells and tissues were washed with 0.1 mol/L phosphate buffer solution (PBS) after treatments and were fixed with 4% formaldehyde. Then, dimethylsulfoxide and 10% goat serum (dissolved in 0.1 mol/L PBS) were used to permeabilize and block the specimens, which were washed again with 0.1 mol/L PBS (0.1% Triton X-100). Anti-ki67 was diluted at the ratio of 1:200 and incubated overnight at 4°C. The secondary antibody incubation was completed with anti-mouse IgG combined with goat anti-rabbit IgG (1:200) and lasted 2 h at room temperature. Finally, the specimens were incubated with diamidino-phenyl-indole (DAPI) solution (5 μg/ml) for 10 min. Using a confocal microscope, images of randomly selected fields (×400 magnification) were acquired.

### TUNEL test

To determine the apoptosis of cells in our research, experiments were conducted in line with the manufacturers' instructions for an apoptosis detection kit.

### Golgi–Cox staining

The morphology of neuronal dendrites and dendritic spines in the brain of mice was observed by Golgi–Cox staining, which was performed with the Hito Golgi–Cox OptimStainTM Prekit (Hitobiotec Corp. Kingsport, TN, USA). Briefly, following the sacrifice of the mice, brain tissues were carefully removed as quickly as possible. Intact brain tissues were rinsed with Milli Q water and impregnated with equal volumes of Solutions A and B from the kit. The impregnation solution was replaced the following day and the tissues were stored at room temperature in the solution in the dark for 2 weeks. Brain tissues were then transferred to Solution C. Solution C was replaced the following day, and brains were further stored at 4°C for 72 h in the dark. Brains were slowly immersed in chilled isopentane for 1 min, wrapped in an aluminum foil, and stored at −70°C until sectioning was performed. Brain sections (100 μm thickness) were generated using a cryostat with the chamber temperature set at −19°C. Each section was mounted on gelatin-coated microscope slides using Solution C. Excess solution was removed from the slide using a Pasteur pipette and further absorbed with stacks of filter paper. These sections were allowed to dry naturally at room temperature (3 days). Dried brain sections were processed as per the manufacturer's instructions. Briefly, dendrites within the CA1 subregion of the hippocampus were imaged using the 20 × and 60 × objectives of an Olympus BX61 applied biosystems. Dendritic spines were detected along the CA1 secondary dendrites starting from their point of origin on the primary dendrite and were counted by an experimenter blind to each sample group.

### Statistical analyses

Statistical analyses were analyzed using the GraphPad Prism version 8.0 statistical package (Graphpad Software, Inc.). Data are presented as mean ± standard error of the mean (SEM). The interaction between time and group factors was determined using a two-way analysis of variance (ANOVA) with repeated measurements to analyze the difference in learning curves between mice in the control or vehicle group and mice in the sevoflurane anesthesia, bicuculline, or nifedipine group in the BM experiments. However, a one-way ANOVA was used in the Barnes maze test, the Y-maze, and the new object recognition experiment. Differences between the groups were assessed with a one-way ANOVA followed by *post hoc* Tukey's multiple comparisons. A significant difference was considered as a *p* < 0.05.

## Results

### Sevoflurane-induced cognitive impairment in young mice and bicuculline or nifedipine can mitigate cognitive impairment caused by sevoflurane

**Figure 2:**
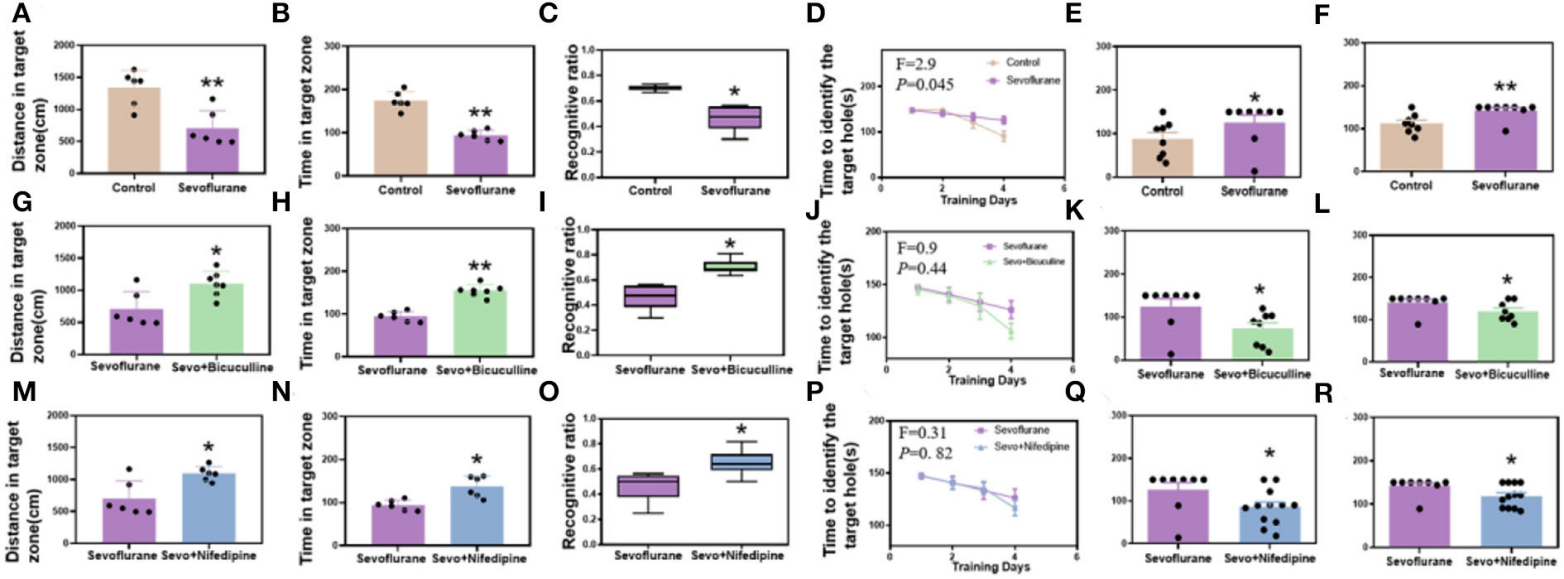
Sevoflurane-induced long-term cognitive dysfunction in mice while bicuculline and nifedipine can relieve cognitive impairment caused by sevoflurane. **(A)** The distance to the target zone traveled by mice in different groups (control vs. sevoflurane, *n* = 6). **(B)** The time mice entered the target zone in different groups (control vs. sevoflurane, *n* = 6). **(C)** New object recognition index in each group of mice (control vs. sevoflurane, *n* = 8). **(D)** The time of target box recognition in the control and sevoflurane groups during the Barnes maze training (control vs. sevoflurane, *n* = 8). **(E)** The time of target box recognition in the Barnes maze short-term memory test (control vs. sevoflurane, *n* = 8). **(F)** The time of target box recognition in the Barnes maze long-term memory test (control vs. sevoflurane, *n* = 8). **(G–L)** Bicuculline group. **(M–R)** Nifedipine group. **(G)** The distance to the target zone traveled by mice in different groups (sevoflurane vs. bicuculline group, Nsevo = 6, Nbicu group = 7). **(H)** The time mice entered the target zone in different groups (sevoflurane vs. bicuculline group, Nsevo = 6, Nbicu group = 7). **(I)** New object recognition index in each group of mice (sevoflurane vs. bicuculline group, *n* = 8). **(J)** The time of target box recognition in the control and sevoflurane groups during Barnes maze training (sevoflurane vs. bicuculline group, *n* = 8). **(K)** The time of target box recognition in the Barnes maze short-term memory test (sevoflurane vs. bicuculline group, *n* = 8). **(L)** The time of target box recognition in the Barnes maze long-term memory test (sevoflurane vs. bicuculline group, *n* = 8). **(M)** The distance to the target zone traveled by mice in different groups (sevoflurane vs. nifedipine group, *n* = 6). **(N)** The time mice entered the target zone in different groups (sevoflurane vs. nifedipine group, *n* = 6). **(O)** New object recognition index in each group of mice (sevoflurane vs. nifedipine group, Nsevo = 8, Nnife = 12). **(P)** The time of target box recognition in the control and sevoflurane groups during Barnes maze training (sevoflurane vs. nifedipine group, Nsevo = 8, Nnife = 12). **(Q)** The time of target box recognition in the Barnes maze short-term memory test (sevoflurane vs. nifedipine group, Nsevo = 8, Nnife = 12). **(R)** The time of target box recognition in the Barnes maze long-term memory test (sevoflurane vs. nifedipine group, Nseco = 8, Nnife = 12). *p < 0.05, **p < 0.01.

Mice were exposed to 3% sevoflurane for 2 h for 3 consecutive days starting from postnatal day 6, and behavioral tests were performed on P56. In the Y-maze, the movement time and distance of the sevoflurane group were significantly lower than those of the control group (*p* < 0.01, Figures 2A,B). In the novel object recognition test, the number of novel object explorations was significantly lower in the sevoflurane group than in the control group (*p* < 0.05, Figure 1C). In the Barnes maze training phase, mice learned the position of the escape box similarly to controls to assess spatial learning and memory. With the increase in training time, the target box recognition time of both groups decreased significantly, and there was a statistical difference between the two groups (*p* < 0.05, Figure 2D). While, in the testing phase, the time of target box recognition was significantly shorter in the control group than in the sevoflurane group, and the difference was statistically significant (*p* < 0.05, Figure 2E, *p* < 0.01, Figure 2F), indicating impaired spatial learning and memory ability of mice in the sevoflurane group.

To explore whether GABRB3 plays a role in sevoflurane-induced cognitive impairment in neonatal mice, we injected bicuculline (a GABRB3 inhibitor) subcutaneously before sevoflurane exposure. In the Y-maze, the movement time and distance of the sevoflurane group were significantly lower than those of the bicuculline group (*p* < 0.01, Figures 2G,H). In the novel object recognition test, the number of novel object explorations was significantly lower in the sevoflurane group than in the bicuculline group (*p* < 0.05, Figure 2I). In the Barnes maze training phase, mice learned the position of the escape box similar to controls to assess spatial learning and memory. With the increase in training time, the target box recognition time of both groups decreased significantly; however, there was no statistically significant difference between the two groups (*p* > 0.05, Figure 2J). While, in the testing phase, the time of target box recognition was significantly shorter in the bicuculline group than in the sevoflurane group, the difference was statistically significant (*p* < 0.05, Figures 2K,L), indicating impaired spatial learning and memory ability of mice in the sevoflurane group.

To investigate whether Cav1.2 plays a role in sevoflurane-induced cognitive impairment in neonatal mice, we subcutaneously injected nifedipine (an inhibitor of Cav1.2) before sevoflurane exposure. In the Y-maze, the movement time and distance were significantly lower in the sevoflurane group than in the nifedipine group (*p* < 0.01, Figures 2M,N). In the novel object recognition test, the number of novel object explorations was significantly lower in the sevoflurane group than in the nifedipine group (*p* < 0.05, Figure 2O). In the Barnes maze training phase, similar to controls, mice learned the position of the escape box to assess spatial learning and memory. With the increase in training time, the target box recognition time of both groups decreased significantly; however, there was no statistically significant difference between the two groups (*p* > 0.05, Figure 2P). While, in the testing phase, the time of target box recognition was significantly shorter in the nifedipine group than in the sevoflurane group, and the difference was statistically significant (*p* < 0.05, Figure 2Q, *p* < 0.05, Figure 2R), indicating the impairment of spatial learning and memory ability of mice in the sevoflurane group.

### Sevoflurane-induced increased the expression of Gabrb3 and Cav1.2 and increased calcium ion concentration in the hippocampus of mice and HT22 cells

**Figure 3:**
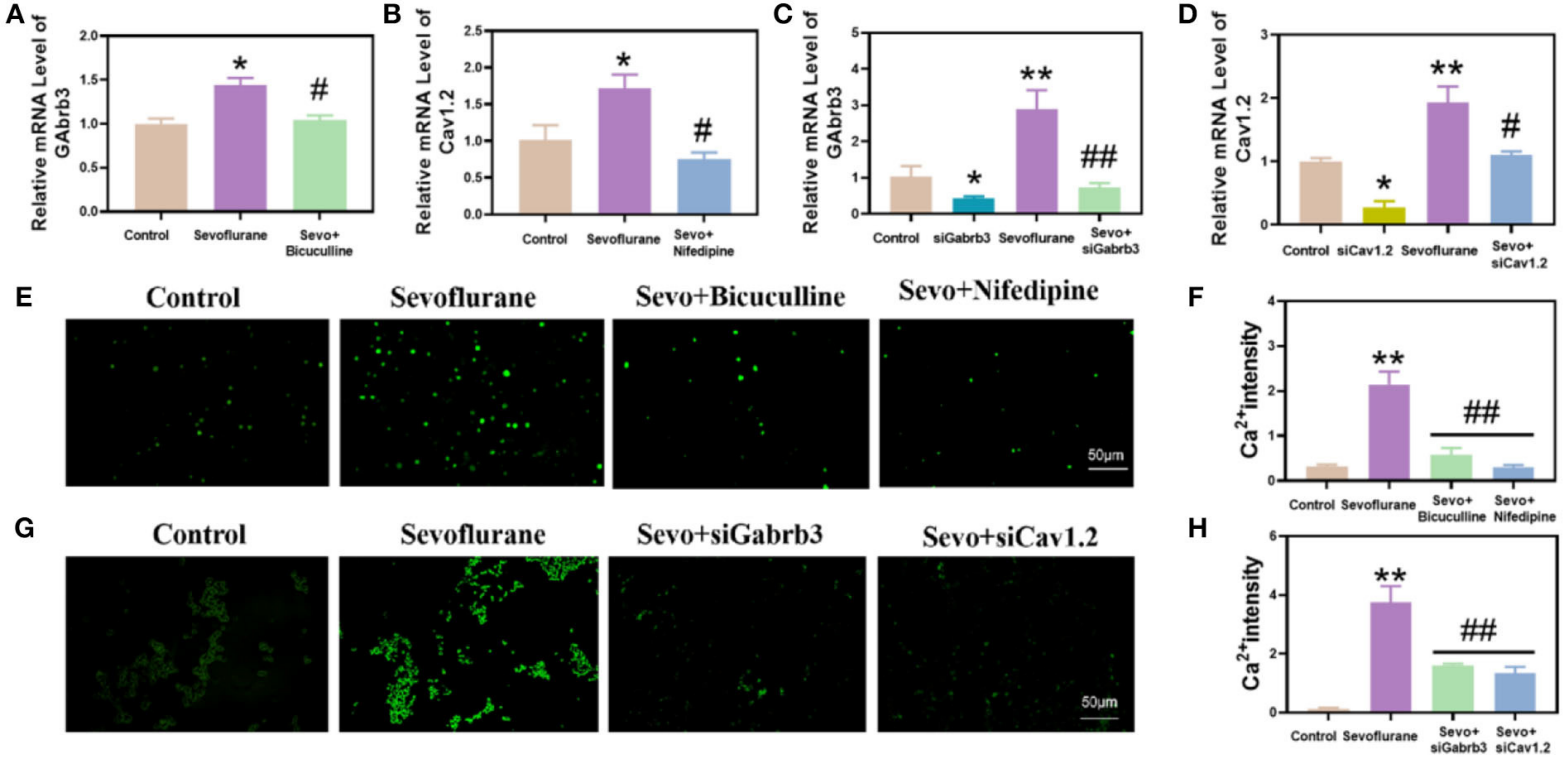
Sevoflurane increased the expression of Gabrb3 and Cav1.2, which resulted in an increase in calcium ion concentration in mouse hippocampal neurons and HT22 cells. **(A)** Gabrb3 in the hippocampus of young mice after various treatments. **(B)** Cav1.2 in the hippocampus of young mice after various treatments. **(C)** Gabrb3 in HT22 cells after various treatments. **(D)** Cav1.2 in HT22 cells after various treatments. **(E)** Fluo-4 calcium ion fluorescence staining of the hippocampus. **(F)** Statistical analysis. **(G)** Fluo-4 calcium ion fluorescence staining of HT22 cells. **(H)** Statistical analysis. **vs. control group, *p* < 0.01, ^#^vs. sevoflurane group, *p* < 0.01, ^##^*p* < 0.01.

Gabrb3 and Cav1.2 were highly expressed in the hippocampus of mice exposed to sevoflurane. Before exposure to sevoflurane, we added bicuculline and nifedipine, respectively, which could effectively inhibit the high expression of Gabrb3 and Cav1.2 (*p* < 0.05, Figures 3A–F). In *in vitro* experiments, Gabrb3 and Cav1.2 were silenced by siRNA. After gene silencing, HT22 cells were exposed to sevoflurane, and the expressions of Gabrb3 and Cav1.2 were also found to be effectively inhibited (*p* < 0.01, Figures 3C,D).

We detected the hippocampal tissue of mice in each group using the fluo-4 calcium fluorescence probe. It was found that the calcium concentration in the hippocampal tissue of mice was significantly higher in the sevoflurane group than in the control group (*p* < 0.01, Figures 3E,F), and the calcium concentration was significantly lower in the bicuculline and nifedipine groups than in the sevoflurane group. At the same time, we also detected the calcium concentration in HT22 cells. This study found that the calcium concentration in HT22 cells was significantly higher in the sevoflurane-treated group than in the control group (*p* < 0.01, Figures 3G,H), and the calcium concentration was significantly lower in the siGabrb3 and siCav1.2 groups than in the sevoflurane group.

### Sevoflurane promoted inflammatory responses in hippocampal neurons and HT22 cells, while bicuculline or nifedipine alleviated sevoflurane-induced inflammation

**Figure 4:**
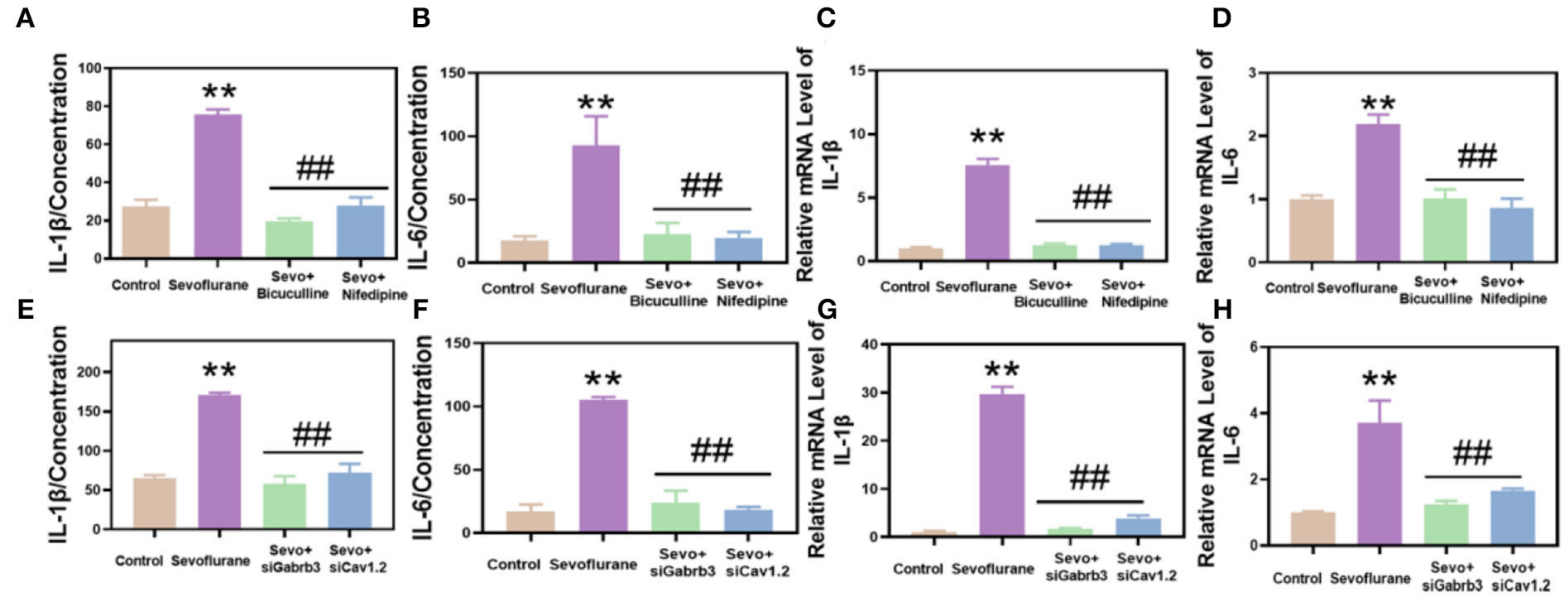
Sevoflurane promoted the inflammatory response of hippocampal and HT22 cells in mice. **(A)** Interleukin-1β (IL-1β) was detected by the enzyme-linked immunosorbent assay (ELISA) method. **(B)** Interleukin 6 (IL-6) was detected by the ELISA method. **(C)** IL-1β was detected by qPCR. **(D)** IL-6 in hippocampal tissues of mice was detected by qPCR. **(E)** IL-1β in HT22 cells was detected by ELISA. **(F)** IL-6 in HT22 cells was detected by ELISA. **(G)** IL-1β was detected by qPCR. **(H)** IL-6 in HT22 was detected by qPCR. ** vs. control group, *p* < 0.01, ^#^vs. sevoflurane group, *p* < 0.05, ^##^*p* < 0.01.

Using RT-qPCR and ELISA methods, the results demonstrated that the inflammatory response of hippocampal neurons in the sevoflurane group was enhanced and the levels of IL-1β and IL-6 were significantly increased, while those in the bicuculline and nifedipine groups were decreased (*p* < 0.01, Figures 4A,B). The results of quantitative real-time PCR (qPCR) were consistent with those of ELISA (*p* < 0.01, Figures 4C,D). The expression levels of IL-1β and IL-6 were significantly decreased. *In vitro* experiments, the inflammatory response of HT22 cells in the same sevoflurane group was significantly increased, while the inflammatory response of HT22 cells was significantly lower in the bicuculline and nifedipine groups than in the sevoflurane group (*p* < 0.01, Figures 4E,F). The results of qPCR were consistent with those of ELISA (*p* < 0.01, Figures 4G,H). Experimental results demonstrated that bicuculline and nifedipine could effectively inhibit sevoflurane-induced inflammatory response.

### Sevoflurane promoted the apoptosis of hippocampal neurons and HT22 cells and inhibited their proliferation, while bicuculline or nifedipine alleviated the effects of sevoflurane

**Figure 5:**
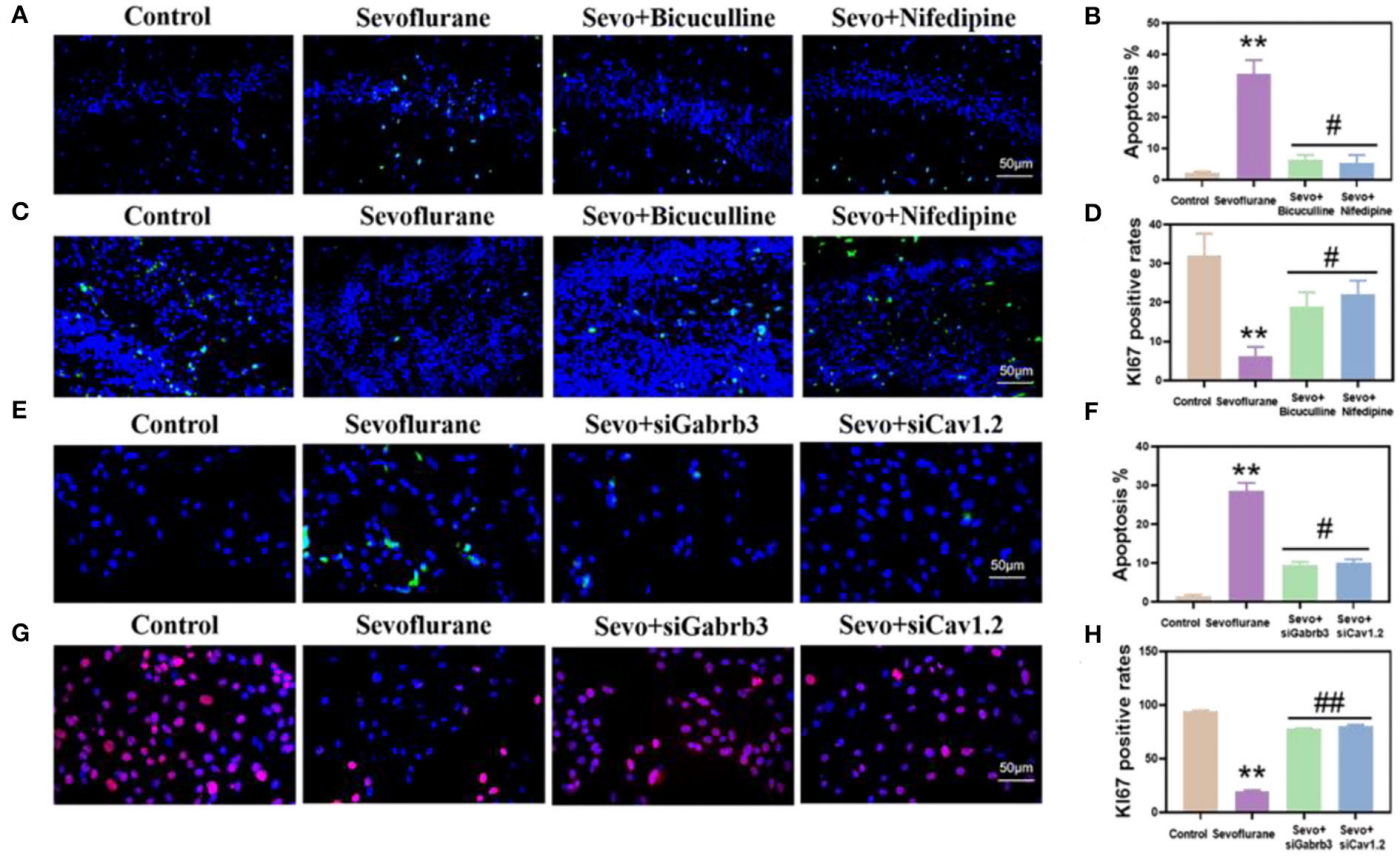
Sevoflurane promoted the apoptosis of hippocampal neurons and HT22 cells and inhibited their proliferation. **(A)** The TUNEL method marked the apoptosis of mouse hippocampal CA1. **(B)** Statistical analysis. **(C)** The proliferation of mouse hippocampal DG labeled by immunofluorescence. **(D)** Statistical analysis. **(E)** Apoptosis of HT22 cells labeled by the TUNEL method. **(F)** Statistical analysis. **(G)** The proliferation of immunofluorescence-labeled HT22 cells. **(H)** Statistical analysis. ** vs. control group, *p* < 0.01, ^#^vs. sevoflurane group, *p* < 0.05, ^##^*p* < 0.01.

Next, we detected the apoptosis and proliferation of hippocampal neurons. The results showed that the apoptosis of hippocampal neurons was significantly higher in the sevoflurane group than in the control group (*p* < 0.01, Figures 5A,B), while the apoptosis of neurons was significantly lower in the bicuculline and nifedipine groups than in the sevoflurane group (*p* < 0.05, Figures 5A,B). The proliferation ability of hippocampal DG neurons in the sevoflurane group decreased (*p* < 0.01, Figures 5C,D), while the proliferation ability of neurons was significantly higher in the bicuculline and nifedipine groups than in the sevoflurane group (*p* < 0.05, Figures 5C,D). In *in vitro* studies, the apoptosis of HT22 cells in the same sevoflurane group significantly increased (*p* < 0.01, Figures 5E,F), while the apoptosis of HT22 cells was significantly lower in the bicuculline and nifedipine groups than in the sevoflurane group (*p* < 0.05, Figures 5E,F). The proliferation ability of HT22 cells was significantly lower in the sevoflurane group than in the control group (*p* < 0.01, Figures 5G,H). The proliferation ability of HT22 cells was significantly higher in the bicuculline and nifedipine groups than in the sevoflurane group (*p* < 0.01, Figures 5G,H).

### Sevoflurane reduced the dendritic spines of hippocampal neurons in mice, and bicuculline or nifedipine reduced the damage caused by sevoflurane

**Figure 6:**
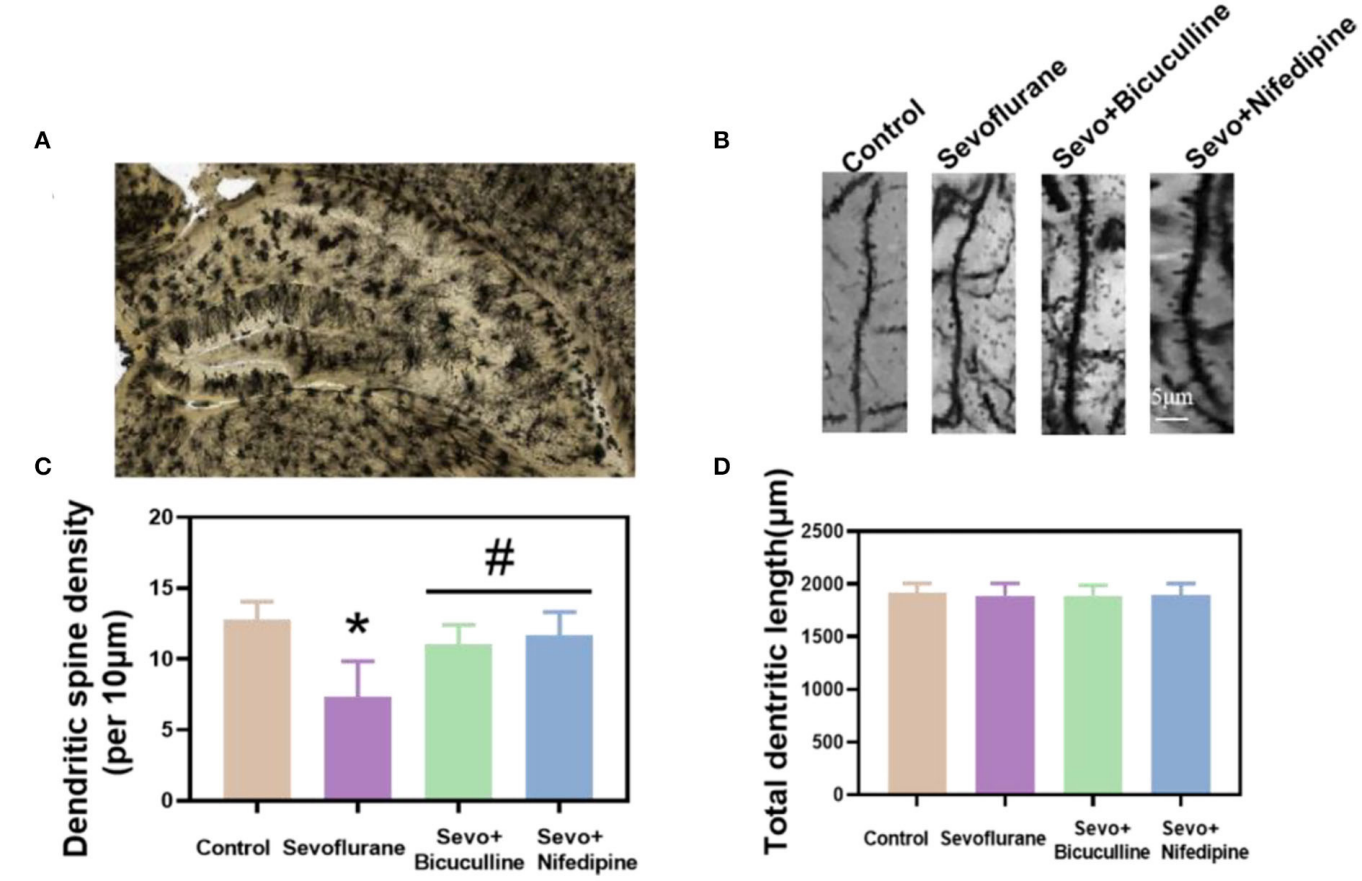
Sevoflurane reduced the density of dendritic spines in mouse neurons. **(A)** A hippocampal profile image of Golgi–Cox staining. **(B)** Density of dendritic spines of hippocampal neurons in mice was stained by Golgi–Cox. **(C)** Statistical analysis. **(D)** Quantitation of the total dendritic length of the five groups. * vs. control group, *p* < 0.05, ^#^vs. sevoflurane group, *p* < 0.05.

To investigate the effects of sevoflurane on the density of dendritic spines in the hippocampus, Golgi staining was used to detect the density of dendritic spines in the hippocampus (Figure 6A) (Luo et al., 2020). The results showed that the density of dendritic spines in the hippocampus of the sevoflurane group was significantly reduced in adult mice (*p* < 0.05, Figures 6B,C), suggesting that sevoflurane affected the density of dendritic spines in the hippocampus of mice. Dendritic spine density in the bicuculline and nifedipine groups was significantly higher than that in the sevoflurane group (*p* < 0.05, Figures 6B,C), indicating that bicuculline and nifedipine could effectively inhibit the decrease in dendritic spine density caused by sevoflurane. There was no significant difference in the total length of dendrites among the groups (*p* > 0.05, Figure 6D).

## Discussion

We performed *in vivo* (mice) and *in vitro* (HT22 cells) studies to explore the potential mechanism of sevoflurane-induced VGCC activation and cognitive impairment. In this study, we found that sevoflurane induces GABA_A_R depolarization and that VGCC activation leads to the accumulation of intracellular calcium ions, which increases neuronal apoptosis, decreases proliferation, increases inflammatory response, and decreases dendritic spine density, and finally leads to long-term cognitive impairment in mice. It is worth noting that the GABA_A_R inhibitor bicuculline and the VGCC inhibitor nifedipine can alleviate these abnormalities. The results demonstrated that GABA_A_R depolarization or VGCC blockade is an effective method of preventing cognitive impairment in mice and that there are possible protective effects of nifedipine on anesthetic neurotoxicity potentially by preventing VGCCs from transporting calcium ions. This study determined the potential underlying mechanism by which sevoflurane induces cognitive impairment in mice.

Previous studies showed that anesthesia with 2.1% sevoflurane concentration for 6 h in P4 rats can cause seizures and the ratio of Na^+^ -K^+^ -Cl^−^ cotransporter (NKCC1)/K^+^ -Cl^−^ cotransporter (KCC2) mRNA (Li et al., 2020). However, bumetanide and bicuculline could mitigate these effects. Neonatal exposure to sevoflurane may cause neurons to adopt a more immature state in which GABA depolarizes and supports neuronal hyperexcitability (Cabrera et al., 2020). In the early stages of embryonic development, depolarizing GABA activates synaptic receptors and regulates neuronal migration. Activation of GABA_A_R in these neurons causes Cl^−^ efflux, strong membrane depolarization or excitation, and Ca^2+^ influx through voltage-gated Ca^2+^ channels and Ca^2+^-permeable N-methyl-D-aspartate receptor channels (Ben-Ari, 2014). GABA_A_R-initiated depolarization and related Ca^2+^ influxes regulate a wide spectrum of biological processes (Ben-Ari, 2014). The magnitude of GABA_A_R excitatory signaling and the proper timing of its transition from excitatory to inhibitory are the keys to normal brain development and function (Hsu et al., 2018). Our results show that bicuculline effectively inhibits the depolarization of GABA_A_R, thereby alleviating the hippocampal inflammatory response and sevoflurane-induced apoptosis of neonatal mice.

Inhaled anesthetics exert anesthetic effects through GABA receptors. Our results show that bicuculline effectively inhibits GABA_A_R depolarization, thereby alleviating sevoflurane-induced hippocampal inflammatory response and apoptosis of neonatal mice. At the same time, it can also alleviate sevoflurane-induced intracellular calcium accumulation and reverse cognitive damage caused by sevoflurane.

Intracellular calcium signaling is an important factor in the regulation of cellular apoptosis (Yamasaki-Mann and Parker, 2011). The intracellular calcium ion balance can be broken under the action of proapoptotic factors, which will lead to sharp increases in intracellular calcium ion concentration. As a result, the sustained increase in intracellular calcium ion concentration will further promote apoptosis. The increase in intracellular calcium ions is mainly derived from the uptake of extracellular calcium ion flow and intracellular calcium store release. Moreover, the voltage dependence of the calcium channel is important in regulating intracellular calcium ions. Intracellular calcium ions interact with calcium-binding proteins to activate an intracellular calcium-dependent protease, leading to cell apoptosis. In addition, the increase in intracellular calcium ions triggers calcium-induced calcium release, showing that high levels of calcium ions will be released from intracellular stores causing intracellular calcium overload and apoptosis.

Voltage-gated calcium channels are transmembrane proteins that are activated in response to cell membrane depolarization and mediate calcium (Ca^2+^) flux into excitatory cells (Heyes et al., 2015; Andrade et al., 2019). An L-type calcium channel is activated and inactivated with a strong depolarizing voltage and a slower time course, respectively, and is expressed in neurons, endocrine, cardiac, and smooth muscles (Dolmetsch et al., 2001). A previous study suggested that long-term exposure to the inhaled anesthetic isoflurane can cause a large influx of Ca^2+^ into nerve cells, resulting in cytotoxicity and a decreased rate of neural progenitor cell proliferation (Zhao et al., 2013). The abnormal state of VGCC can lead to various diseases, such as epilepsy and Alzheimer's disease. Meanwhile, cerebral ischemia and hypoxia cause an abnormal VGCC state (Kim, 2015). Our study shows that exposure to sevoflurane causes the activation of VGCCs and increases calcium ion concentration in neurons, while the addition of the inhibitor nifedipine effectively reduces calcium ion concentration in neurons. Nifedipine also reversed sevoflurane-induced neuronal apoptosis and inflammatory response as well as cognitive impairment in mice.

In conclusion, our results suggest that neurotoxicity due to calcium ion accumulation in neurons caused by VGCCs is one of the potential mechanisms of sevoflurane-induced long-term cognitive dysfunction in neonatal mice. Sevoflurane-induced increase in GABAAR depolarization activates VGCCs. Accumulation of Ca^2+^ ions in neurons leads to cognitive impairment in neonatal mice. These findings pave the way for further research on the effects of anesthesia on the developing brain.

## Data availability statement

The raw data supporting the conclusions of this article will be made available by the authors, without undue reservation.

## Ethics statement

The animal study was reviewed and approved by Grass-roots Ethics Review Committee of Zhengzhou University.

## Author contributions

Conception and design: SZ, RZ, YW, YY, NL, NF, MS, and JZ. Collection and assembly of data: SZ, MS, and JZ. Data analysis and interpretation: SZ and JZ. All authors contributed to the article and approved the submitted version.

## Funding

SZ and JZ received funding from the National Natural Science Foundation of China (82071217, 81771149, and 81901110) and the Natural Science Foundation for Outstanding Youth of Henan province (212300410073).

## Conflict of interest

The authors declare that the research was conducted in the absence of any commercial or financial relationships that could be construed as a potential conflict of interest.

## Publisher's note

All claims expressed in this article are solely those of the authors and do not necessarily represent those of their affiliated organizations, or those of the publisher, the editors and the reviewers. Any product that may be evaluated in this article, or claim that may be made by its manufacturer, is not guaranteed or endorsed by the publisher.
